# An Essay on the Chemical, Botanical, Physical, and Parturient Properties of the Secale Cornutum: With an Engraving

**Published:** 1841-10

**Authors:** 


### ART. XVIII.

*An Essay on the Chemical, Botanical, Physical, and Parturient Properties of the Secale Cornutum: with an Engraving.* By T. H. WARDLEWORTH, Surgeon.—*London*, 1840. 12mo, pp. 69.

THE purport of this book is to increase the confidence of the profession in the "parturifacient" effects of the secale cornutum. As a proof of the author's zeal in the cause he advocates, we quote the following passage:

"The efforts of the profession will doubtless, ere long, ascertain the full merits of the medicine in question, and if our expectations are realized, will place the secale cornutum amongst the most important discoveries in medicine, and mark the age in which its merits were established in the same page with those that record the discovery of the VACCINE VIRUS." (p. 35.)

To us, the comparison which is here so clumsily instituted, seems to be quite absurd, but we will state the grounds upon which Mr. Wardleworth founds his very high opinion of the "secale cor,"—for thus does he, in his prescriptions, deprive his favorite agent of its fair abbreviated proportions.

"In the course of my practice the secale cornutum has been administered to 1500 patients, without selecting or in any way giving a preference to any individual case, or professing that knowledge by which a partiality might have been given, for so little has been published on the subject, that, in common with other practitioners, feelings of hesitation have sometimes passed in my mind when administering a drug of which nothing was known but the general character of its common effects. Yet under these circumstances I have administered it to 1500 patients, as before mentioned, with the most satisfactory results: and that a mode of treatment which has proved so safe, and so salutary, and which to a great extent has lessened the period of suffering, may become a great object to medical practitioners, this attempt to excite more general attention to the subject has been made." (p. 32.)

Now we submit to Mr. Wardleworth, that such a statement as this is just as little adapted to lead to the conclusion he wishes, as it is to prove either the elegance or the accuracy of his composition. We will not insinuate even a suspicion of its accuracy as to facts; still the number of patients mentioned is very considerable, and shows both the vast extent of Mr. Wardleworth's practice and the exuberant fertility of the ladies in and about "Rochdale." But it does not show either the necessity or the propriety of having thus administered the "secale cor." And we declare it to be our belief, that 1500 cases of labour have never fallen to the share of any practitioner, in which the use of the ergot of rye, or any other "parturifacient" agent was either required or justifiable. We have not the smallest doubt that, in the great majority of these 1500 cases, in which "without selecting, or in any way giving a preference to any individual case," this "partûs accelerator" was given, that the efforts of nature might, and ought to have been relied upon. Mr. Wardleworth quotes Chevreul, who, we are told, is of opinion that the use of the secale cornutum "is applicable in the early stages of all natural labours;" "an opinion with which I (Mr. Wardleworth) fully concur, provided the patient has, in the last months of pregnancy, submitted to a prescribed diet, and if advised has taken a few doses of medicine and lost a few ounces of blood: by these means, simple as they may appear, the sorrow and danger of child-birth will be greatly lessened, and its duration the subject, not of uncertainty, but of calculation," (p. 41.) We have no fear that others will take to this mischievous and meddlesome midwifery, and we strongly advise Mr. Wardleworth to allow his "feelings of hesitation" to have a little more influence upon his practice, and that in at least some part of his next 1500 cases, he will give nature fair play, and we venture to prophecy that he will find "natural labours" do not require the impertinent interference which he so unwisely and so injudiciously recommends.

The extracts we have given are sufficient samples of Mr. Wardleworth's loose and very inaccurate style of writing; but many of the blunders we have detected we attribute, in charity, to the printer: as for example, "Desmoreaux" for Desormeaux: "per vaginum:" "dilitation" of the os uteri, "agrum" for ægrum, &c. &c.

